# Comprehensive Analysis of Inflammatory Immune Mediators of the Intraocular Fluid Aspirated from the Foldable Capsular Vitreous Body Filled-Eyes

**DOI:** 10.1371/journal.pone.0046384

**Published:** 2012-10-01

**Authors:** Peijuan Wang, Qianying Gao, Xiaofeng Lin, Shaochong Zhang, Jie Hu, Yaqin Liu, Nuo Xu, Jian Ge

**Affiliations:** 1 State key Laboratory of Ophthalmology, Zhongshan Ophthalmic Center, Sun Yat-sen University, Guangzhou, China; 2 Department of Ophthalmology, Fujian Provincial Hospital, Fujian Provincial Clinical College of Medicine, Fuzhou, China; Wayne State University, United States of America

## Abstract

**Purpose:**

To analyze the level of human inflammatory immune mediators in the intraocular fluid aspirated from foldable capsular vitreous body (FCVB) filled-eyes during FCVB removal surgery, 3 months after implantation.

**Methods:**

8 samples of intra-FCVB fluids (n = 8) were collected from 8 FCVB filled patients in our previous FCVB exploratory clinical trial. The intra-FCVB fluids were aspirated from the FCVB filled-eyes during the FCVB removal surgeries at the third month. For the control groups, the vitreous fluids were collected from patients with idiopathic macular hole (n = 9) or rhegmatogenous retinal detachment (n = 6) during pars plana vitrectomy. A multiplex immunoassay was used to determine levels of 9 cytokines (IL-1β, IL-2, IL-6, IL-8, IL-10, IL-17, TNF-α, IFN-γ and VEGF) in these samples. The VEGF level of some intra-FCVB fluids (n = 6) were re-tested using enzyme-linked immunosorbent assay (ELISA).

**Results:**

In the intra-FCVB fluids, 9 cytokines concentrations of most samples (n = 5) measured by Multiplex immunoassay showed low values, except for Patient 02, 06, and 09. The VEGF concentrations of some intra-FCVB fluids (n = 6) tested by ELISA were in accordance with Multiplex immunoassay results. For all eight patients (n = 8), the concentrations of IL-1β, IL-2, IL-6, TNF-α, IFN-γ and VEGF were slightly higher as compared to the idiopathic macular hole control group. While, the concentrations of IL-8, IL-10, and IL-17 were not statistically significant different compared with the idiopathic macular hole control samples. Most cytokines concentrations (IL-2, IL-6, IL-8, IL-10, IL-17, TNF-α, IFN-γ, VEGF) were not statistically significant different compared to the rhegmatogenous retinal detachment control group except IL-1β.

**Conclusions:**

The FCVB had sufficient porosity to allow cytokines to pass through. This study first discovered that the FCVB possesses favorable permeability of proteins in the human eye.

## Introduction

Artificial vitreous substitutes are crucial therapy tools to assist in the treatment of severe retinal detachments caused by proliferative diabetic retinopathy (PDR), proliferative vitreoretinopathy (PVR), traumatic PVR, and endophthalmitis [1]–[7]. This area has become one of the most studied topics in the field of ophthalmology, and there is much data regarding the characteristics of artificial vitreous substitutes, both in vitro and in vivo [8]–[10].

Many different types of artificial vitreous substitutes has so far been studied mainly in the following categories: gas (air and expansile gas), liquid (balanced salt solution, perfluorocarbon liquid, semi-fluorinated alkanes and silicone oil), natural polymers (hyaluronic acid), semi-synthetic polymers (“hylan”) and synthetic polymers (hydrogels and smart hydrogel). Some of them have been widely used in clinical practice, and some of them even have been in the experimental stage [8]–[10]. Silicone oil is one of the most common vitreous substitutes in the treatment of severe retinal detachment, and many studies have shown that silicone oil is a relatively safe vitreous substitute. In clinical practice, however, silicone oil complications have been observed, including glaucoma, cataract, corneal degeneration, and silicone oil emulsification [11]–[13]. The problems still exist such as severe inflammatory reactions and retinal damage secondary to silicone oil when heavy silicone oil appears in clinic [14]–[17]. Therefore, the search for an ideal artificial vitreous substitute and materials continues.

In our previous studies, we reported our invention of a novel foldable capsular vitreous body (FCVB) [18]–[22]. Reports from the State Food and Drug Administration in China showed that the FCVB has good mechanical, optical, and biocompatible properties (No. G20080656) [20]. The FCVB has never been used in human eyes, therefore, we conducted an exploratory study of 11 patients who had a FCVB implanted as part of the surgical treatment of severe retinal detachment at Zhongshan Ophthalmic Center. The study found that the FCVB had good flexibility, safety, and efficacy during a three-month study period [22]. We then collected fluids from FCVB-filled eyes during FCVB removal surgery at the third month, and analyzed the cytokines to learn whether the composition of the intra-FCVB fluid changed over time, and whether these low-molecular-weight proteins could enter into the FCVB. We chose following 9 cytokines: IL-1β, IL-2, IL-6, IL-8, IL-10, IL-17, TNF-α, IFN-γ and VEGF. These cytokines usually appeared during inflammatory processes on wound repair after surgery.

## Patients and Methods

### Study Design

This study was a part of the FCVB exploratory clinical trial. The study protocol of this clinical trial was reviewed and approved by the Sun Yat-sen University Medical Ethics Committee (Zhongshan Ophthalmic Center Medical Ethics [2009] No. 07). The clinical trials strictly adhere to the principles of The World Medical Association Declaration of Helsinki and have been successfully registered with ClinicalTrials.gov (ClinicalTrials.gov ID: NCT00910702) as well as the Chinese Clinical Trial Register (ChiCTR-TNC-00000396). All patients gave written informed consent [22].

Patients (11 eyes) with severe retinal detachments were recruited for this clinical trial between May 2009 and January 2010 according to the following inclusion and exclusion criteria. The inclusion criteria were 1) severe posterior scleral ruptures with large retinal absence, 2) severe scleral ruptures with retinal detachments and choroidal detachments, and 3) rigid retinal re-detachments or inferior holes after silicone oil or heavy silicone oil tamponade. Exclusion criteria were 1) patients with serious heart, lung, liver, or kidney dysfunction, 2) patients with other ocular diseases including autoimmune uveitis, PDR, pathological myopia and so on, 3) patients with a single eye, 4) patients with stable silicone oil-filled eyes, and 5) patients with diseases that the researchers deemed them unsuitable for participation in this clinical trial.

Nine idiopathic macular hole patients (9 eyes) and six rhegmatogenous retinal detachment patients (6 eyes) were also recruited as the control groups, and undiluted vitreous fluids from these eyes were collected. Eyes with significant PVR or patients with systemic diseases including diabetes mellitus, hypertension, immunological diseases, and infectious diseases were excluded.

### Study Treatment

**Figure 1:**
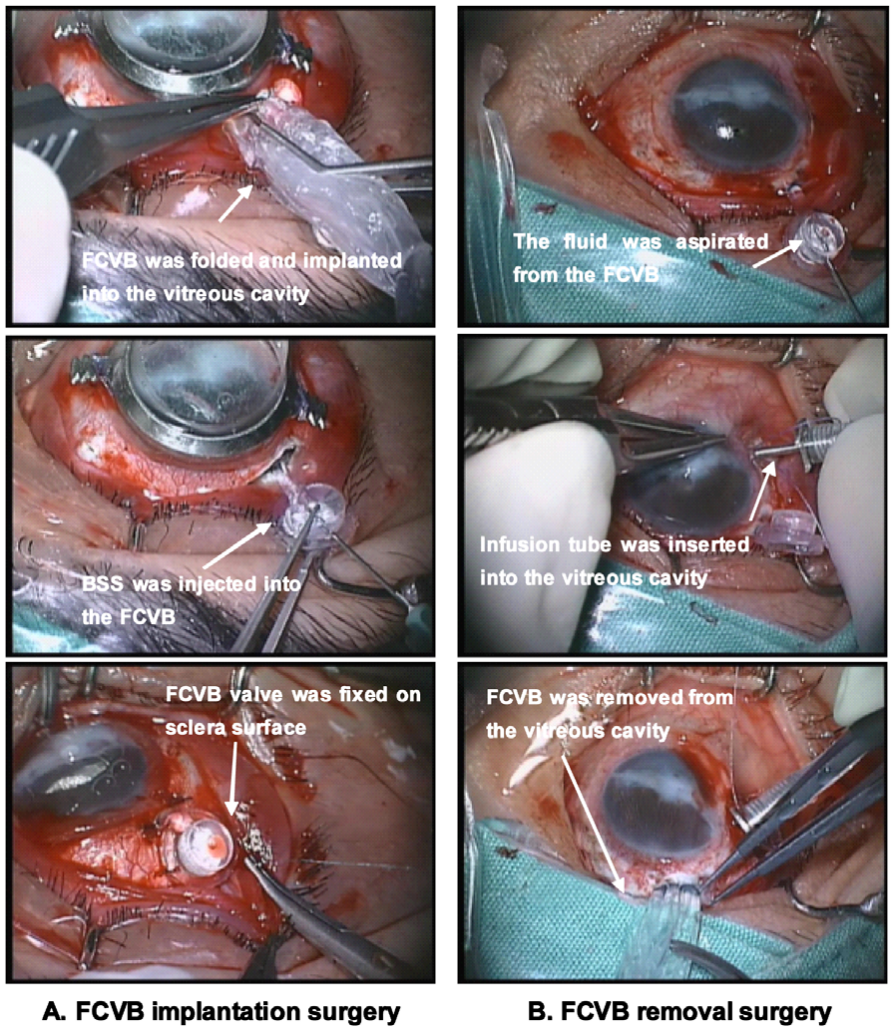
The implantation and removal surgery of FCVB on the same eye of Patient 04. **A. FCVB implantation surgery:** The FCVB was triple-folded and implanted into the vitreous cavity. Approximately 4.0 ml of BSS was injected into the capsule to support the retina. The valve and tube were subsequently fixed on the scleral surface. **B. FCVB removal surgery:** The fluid inside the FCVB was aspirated by a 2 ml-syringe, prior to intraocular infusion of BSS. The capsule was cut as needed to allow FCVB removal.

Intervention procedures consisted of vitrectomy, FCVB (Guangzhou Weishibo Co, Ltd, Guangzhou, China) implantation, and FCVB removal three months later. The procedure of FCVB implantation surgery was described in detail in our previous exploratory study of 11 FCVB filled-eyes. In FCVB implantation surgery, approximately 4.0 ml of balance salt solution (BSS) was injected into the FCVB through the valve, to provide support for the retina (Figure 1A). Ocular examinations were performed at baseline and day 3, week 1, 2, 4, 6, and 8, and 3 months after FCVB implantation.

After 3 months of FCVB implantation, intra-FCVB fluid (0.2–0.7 ml) was first collected during FCVB removal surgery. The conjunctiva was dissected from 10∼2 o'clock to expose the FCVB valve thoroughly. The fluid inside the FCVB was aspirated by a 2 ml-syringe, prior to intraocular infusion of BSS (Figure 1B). All samples were immediately transferred into sterile 1.5 ml polypropylene tubes on ice and were centrifuged at 10000 g for 10 minutes at −4°C, aliquoted, and stored at −80°C until assayed.

Undiluted vitreous fluid (0.2–0.7 ml) was then collected from the patients with idiopathic macular hole or rhegmatogenous retinal detachment. A 23G/20G three-port pars plana vitrectomy was set up, and vitreous samples were obtained from the mid-vitreous using a vitreous cutter, prior to intraocular infusion of BSS. These vitreous samples were transferred directly into sterile polypropylene tubes on ice and subsequently centrifuged at 10000 g for 10 minutes at −4°C, aliquoted, and stored at −80°C until assayed.

### Multiplex Immunoassay

The concentrations of cytokines in the intra-FCVB fluid and vitreous fluid were analyzed by Multiplex immunoassay according to the manufacturer's instructions. A Human MultiAnalyte Profiling Base Kit A, Cat. No. LUH000 (R&D System, Inc. Minneapolis, USA) was used and the following nine cytokines were measured: IL-1β, IL-2, IL-6, IL-8, IL-10, IL-17, TNF-α, IFN-γ and VEGF.

All samples were repeatedly measured 3 times. First, color-coded microparticles (pre-coated with analyte-specific antibodies), protein standards in a known concentration and test samples (intra-FCVB fluid and undiluted vitreous fluid) were pipetted into wells of a filter-bottom microplate. Incubating 3 hours at room temperature (RT). After that, washing away any unbound substances. Then, a biotinylated antibody cocktail specific to the analytes of interest was added to each well. Incubating 1 hour at RT. Following a wash to remove any unbound biotinylated antibody, streptavidin-phycoerythrin conjugate (Streptavidin-PE), which bound to the biotinylated detection antibodies, was added to each well. Incubating 30 minutes at RT. Last, a final wash removed unbound Streptavidin-PE and the microparticles were resuspended in buffer and read using the Luminex 200 analyzer. All incubation processes were performed on a horizontal orbital microplate shaker.

### Enzyme-linked Immunosorbent Assay (ELISA)

The VEGF level of intra-FCVB fluids from 6 study patients (Patient 02, 03, 05, 07, 09, and 11) was measured using a Human VEGF Immunoassay Kit, Cat. No. DVE00 (R&D System, Inc. Minneapolis, USA) according to the manufacturer's instructions. Other samples could not be analyzed by ELISA, due to volume limitation.

All samples were measured twice. This assay employed the quantitative sandwich enzyme-linked immunoassay technique. First, VEGF standards and test samples (intra-FCVB fluid) were pipetted into wells of a microplate pre-coated with a monoclonal antibody specific for VEGF. Incubating 2 hours at RT. After washing away any unbound substances, an enzyme-linked polyclonal antibody specific for VEGF was added into the wells. Incubating 2 hours at RT. Following a wash to remove any unbound antibody-enzyme reagent, a substrate solution was added into each well and color developed in proportion to the amount of VEGF bound in the initial step. The color development was stopped and the VEGF concentration was read using the Microplate Reader.

### Statistical Analysis

The concentrations of all cytokines were described as mean ± standard deviation (SD). Two-group comparisons of each cytokine concentration was done based on the distribution pattern of data using the Two Independent Sample T-test or the Mann-Whitney Test. Statistical significance was considered to be present by a P value<0.05 in a two-tailed test.

## Results

### Study patients

After 3 months of FCVB implantation, retinal reattachments were found in 8 FCVB filled-eyes, while retinas were redetached in other 3 eyes because of the FCVB capsule leakage during FCVB implantation period. Thus, the intra-FCVB fluids were obtained from only above 8 patients (n = 8) during FCVB removal surgery. The demographic and ocular characteristics of these 8 patients at baseline examination are shown in Table 1.

**Table 1:** Demographic and ocular Characteristics of patients with FCVB implantation.

Patient	Age (Y)	Gender	History of surgery	Diagnosis
02	13	Male	1. Corneal wound suturing+ formation of anterior chamber; 2. PPV+ C_3_F_8_	**Penetrating ocular injury (OS)** 1. Penetrating corneal trauma 2. Hyphema 3. Iridocoloboma 4. Aphakic eye 5. Vitreous opacity 6. Retinal detachment
03	19	Male	1. Corneal wound suturing; Lensectomy+ PPV+ silicone oil	**Ocular contusion (OD)** 1. Corneal laceration 2. Iridocoloboma 3. Aphakic eye 4. Silicone oil eye 5. Retinal detachment
04	38	Male	1. Corneal wound suturing	**Penetrating ocular injury (OD)** 1. Penetrating corneal trauma 2. Ttraumatic cataract 3. Retinal detachment
05	13	Male	1. Corneal wound suturing; 2. PPV+ silicone oil	**Ocular contusion (OD)** 1. Corneal laceration 2. Iridocoloboma 3. Aphakic eye 4. Silicone oil eye
06	30	Female	1. Corneal wound suturing; 2. Lensectomy+ PPV; 3. Encircling scleral buckling+ PPV+ silicone oil	**Penetrating ocular injury (OD)** 1. Penetrating corneal trauma 2. Aphakic eye 3. Silicone oil eye 4. Retinal detachment
07	43	Male	1.Scleral wound exploration and suturing+eye skin laceration suture	**Ocular contusion (OS)** 1. Scleral laceration 2. Traumatic cataract 3. Vtreous hemorrhage 4. Choroidal detachment 5. Retinal detachment
09	25	Male	1. Intraocular foreign body extraction; 2. PPV; 3. PPV+ C_3_F_8_; 4. PPV+ heavy silicone oil	**Penetrating ocular injury (OS)** 1.Penetrating scleral trauma 2. Traumatic cataract 3. Heavy silicone oil eye 4. Rretinal detachment
11	30	Male	1. Corneoscleral trauma suturing	**Penetrating ocular injury (OS)** 1. Penetrating corneoscleral trauma 2. Traumatic cataract 3. Choroidal detachment 4. Retinal detachment

The demographic and ocular characteristics of the two control groups, including idiopathic macular hole patients (n = 9, M1∼M9) and rhegmatogenous retinal detachment patients (n = 6, D1∼D6), are shown in Table 2.

**Table 2:** Demographic and ocular Characteristics of patients in control groups.

Patient	Age(Y)	Gender	History of surgery	Diagnosis
M1	63	Male	None	1. Age related cataract (OU)2. Idiopathic macular hole (OD)
M2	63	Female	None	1. Idiopathic macular hole (OD)
M3	63	Female	None	1. Idiopathic macular hole (OS)
M4	57	Female	None	1. Idiopathic macular hole (OS)
M5	60	Female	None	1. Idiopathic macular hole (OD)
M6	56	Female	None	1. Idiopathic macular hole (OS)
M7	66	Female	None	1. Idiopathic macular hole (OD)
M8	54	Female	None	1. Idiopathic macular hole (OS)
M9	46	Female	None	1. Idiopathic macular hole (OD) 2. Refractive error (myopia) (OU)
D1	76	Female	None	1. Age related cataract (OS) 2. Rhegmatogenous retinal detachment (OS)
D2	76	Male	Phacoemulsification+IOLimplantation	1. IOL eye (OD) 2. Rhegmatogenous retinal detachment (OD)
D3	28	Female	None	1. Rhegmatogenous retinal detachment (OS) 2. Retinal lattice degeneration (OS) 3. Refractive error (myopia) (OS)
D4	55	Female	None	1. Rhegmatogenous retinal detachment (OD)
D5	39	Male	None	1. Rhegmatogenous retinal detachment (OD) 2. Refractive error (myopia) (OU)
D6	41	Male	None	1. Rhegmatogenous retinal detachment (OS)

M1∼M9 were 9 idiopathic macular hole patients.

D1∼D6 were 6 rhegmatogenous retinal detachment patients.

### Ocular status of FCVB filled-eyes during FCVB implantation period

Before the FCVB removal surgery, ocular examinations were performed on FCVB filled-eyes, including visual acuity (VA), intraocular pressure (IOP), slit lamp biomicroscopy, fundus photography, noncontact specular microscopy, B-scan ultrasonography, optical coherence tomography (OCT), and ultrasound biomicroscopy (UBM). VA and IOP in the FCVB treated-eyes showed no significant difference compared with the preoperative measurements. The fundus photography, B-scan, and OCT showed that the FCVB was evenly supported the retina. No corneal keratopathy, glaucoma or uncontrollable intraocular inflammation was observed, and no adverse events (e.g., unbearable foreign body sensations, abnormal bleeding, severe inflammation, endophthalmitis, or sympathetic ophthalmia) was observed during this clinical trial. No corneal edema, keratic precipitates, aqueous flare or aqueous cell was observed and only slight conjunctival chemosis was observed in a few FCVB filled-eyes after three months [22].

**Figure 2:**
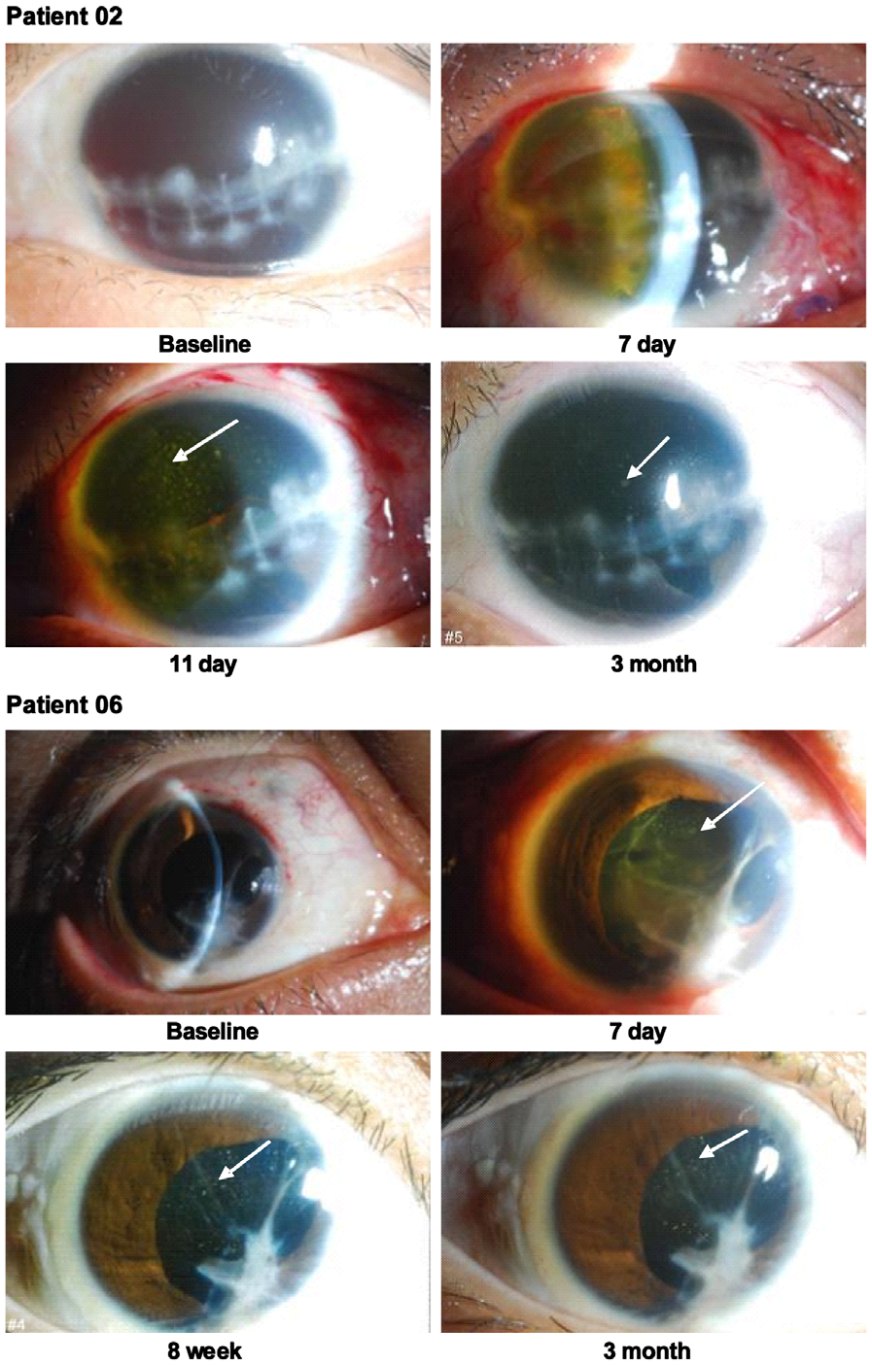
Anterior segment status of FCVB-filled eyes at baseline and at FCVB implantation. Patient 02 and 06 both suffered from severe ocular injury. Patient 02 had a marked hyphema and exudation in the anterior chamber in the first week after FCVB implantation. At the 11th day, a few point-like opacities (white arrow) were found in the intra-FCVB fluid and these opacities persisted until the 3th month. Patient 06 also had a few point-like opacities in the intra-FCVB fluid in the 7th day, and these opacities was persisted until the 3th month. However, no anterior segment inflammation (conjunctival chemosis, corneal edema, keratic precipitates, aqueous flare or aqueous cell) was found in these two patients after three months.

Unexpectedly, after FCVB implantation surgery, a few point-like opacities were found in the intra-FCVB fluid in Patient 02 and 06 on day 11 and day 7, respectively. These opacities persisted until the 3th month in the FCVB in these two patients, while no anterior segment inflammation (conjunctival chemosis, corneal edema, keratic precipitates, aqueous flare or aqueous cell) was found in these two patients after three months (Figure 2). Laboratory examinations (HE staining) showed that these opacities were a few translucent membranes in the intra-FCVB fluids and there were no inflammatory cells, bacteria, or fungi present [22].

### Nine cytokines concentrations measured by Multiplex Immunoassay

In the control group of idiopathic macular hole, the concentrations of all 9 cytokines showed low values. In another control group of rhegmatogenous retinal detachment, the concentrations of most cytokines showed low values except IL-6, IL-8, and VEGF (Table 3).

**Table 3:** 9 cytokines concentrations measured by Multiplex Assay in control groups (pg/ml).

Patient	IL-1β	IL-2	IL-6	IL-8	IL-10	IL-17	TNF-α	IFN-γ	VEGF
M1	0.63	<OOR	1.26	2.06	1.16	1.10	1.36	1.99	4.33
M2	0.20	<OOR	0.22	0.42	0.83	<OOR	1.31	1.48	0.56
M3	<OOR	<OOR	8.66	70.54	0.75	<OOR	1.26	1.51	16.08
M4	0.20	<OOR	0.32	0.29	0.72	<OOR	1.31	1.26	0.38
M5	0.41	<OOR	0.22	0.86	0.67	<OOR	1.07	1.54	0.74
M6	0.31	<OOR	0.64	0.65	1.00	0.66	1.21	2.27	1.87
M7	0.20	<OOR	0.22	0.37	0.83	<OOR	1.31	1.54	0.50
M8	0.48	<OOR	1.06	3.00	0.91	<OOR	1.17	1.48	1.67
M9	0.41	<OOR	1.26	13.58	0.61	<OOR	1.26	1.88	0.56
Mean	0.355	—	1.540	2.654	0.832	0.880	1.251	1.663	2.961
SD	0.156	—	2.707	4.518	0.172	0.311	0.090	0.317	5.077
D1	0.20	<OOR	13.95	17.00	0.83	<OOR	0.97	1.60	0.91
D2	0.41	<OOR	51.39	87.49	0.83	<OOR	1.26	1.37	2.96
D3	0.34	<OOR	13.76	38.84	1.00	<OOR	1.17	1.82	10.15
D4	1.78	1.28	51.45	64.27	0.83	0.66	2.52	5.87	70.67
D5	1.81	0.66	62.24	156.46	0.69	0.55	2.28	5.80	1867.17
D6	1.78	1.22	4.38	77.27	0.60	0.87	2.40	5.91	9.34
Mean	1.053	1.053	32.860	73.555	0.797	0.693	1.767	3.728	326.866
SD	0.890	0.342	24.842	48.107	0.138	0.163	0.705	2.340	755.045

M1∼M9 were 9 idiopathic macular hole patients.

D1∼D6 were 6 rhegmatogenous retinal detachment patients.

<OOR="Below and Out Of Range”.

**Figure 3:**
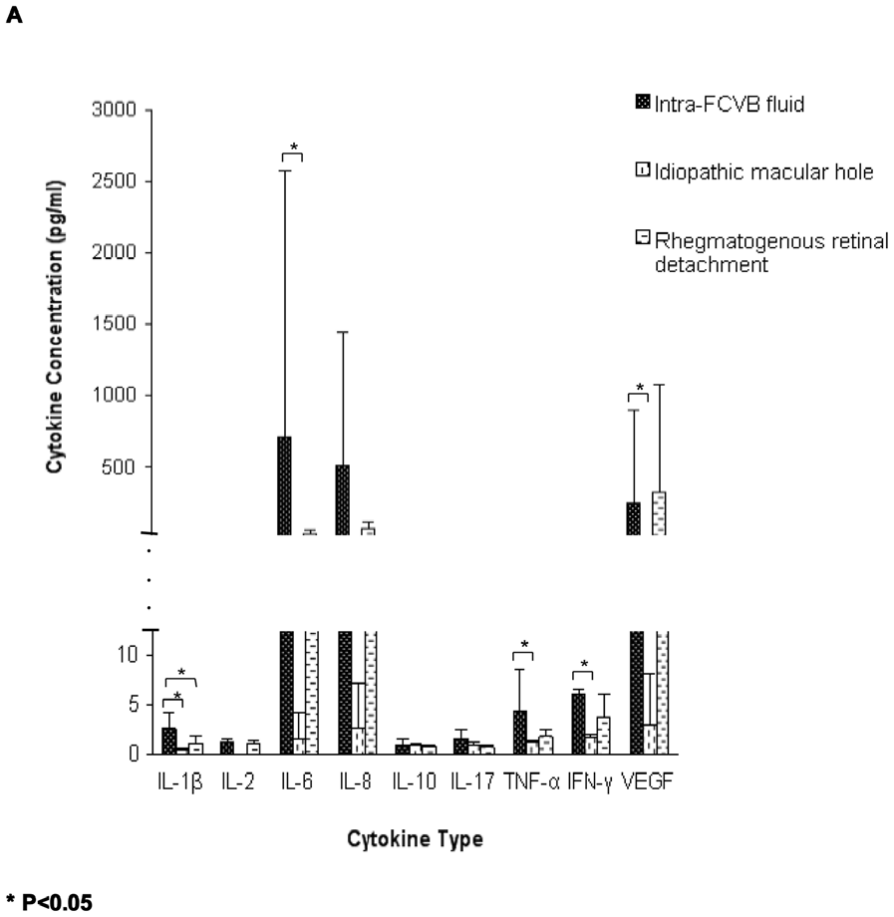
Comparison of nine cytokine concentrations between the intra-FCVB fluid (n = 8) and the control groups measured by Multiplex Immunoassay. Compare to the idiopathic macular hole control group, the concentrations of IL-1β, IL-2, IL-6, TNF-α, IFN-γ and VEGF were slightly higher in the intra-FCVB fluid. (IL-2 was below the range of detection sensitivity for samples from idiopathic macular hole patients). The concentrations of IL-8, IL-10 and IL-17 were not statistically significant different compared with the idiopathic macular hole patients. Compared to the rhegmatogenous retinal detachment control group, most cytokines concentrations in the intra-FCVB fluid were not statistically significant different, except IL-1β.

Intra-FCVB fluid analysis of 9 cytokines concentrations of most samples measured by Multiplex immunoassay showed low values except for Patient 02, 06, and 09 (Table 4). For all eight patients (n = 8), the concentrations of IL-1β, IL-2, IL-6, TNF-α, IFN-γ and VEGF were slightly higher as compared to the idiopathic macular hole control group. The concentrations of IL-8, IL-10, and IL-17 were not statistically significant different compared with the idiopathic macular hole control samples (Mann-Whitney Test: IL-1β P = 0.001; IL-6 P = 0.033; IL-8 P = 0.847; IL-10 P = 0.385; IL-17 P = 0.428; TNF-α P = 0.001; IFN-γ P = 0.001; VEGF P = 0.002; IL-2 had no P value because the value of IL-2 was below the range of detection sensitivity for the vitreous samples from idiopathic macular hole control patients) (Figure 3).

**Table 4:** 9 cytokines concentrations measured by Multiplex Assay in intra-FCVB fluid (pg/ml).

Patient	IL-1β	IL-2	IL-6	IL-8	IL-10	IL-17	TNF-α	IFN-γ	VEGF
02	3.40	0.72	5307.19	1783.83	1.70	0.85	3.69	5.8	1864.25
03	1.83	1.37	1.22	0.23	0.42	0.74	2.18	5.77	8.48
04	1.78	1.27	1.22	0.27	0.51	0.77	2.12	5.82	8.42
05	1.78	1.27	1.22	0.27	0.51	0.77	2.12	5.82	8.42
06	6.43	1.24	402.87	2226.58	1.68	2.85	14.22	5.82	37.44
07	1.78	1.41	1.35	7.45	1.53	2.31	6.45	5.8	25.35
09	1.93	1.10	4.57	121.47	0.51	0.77	2.15	5.83	27.2
11	1.78	1.84	1.22	0.21	0.40	2.93	2.21	7.24	8.56
Mean	2.589	1.278	715.108	517.539	0.908	1.499	4.393	5.988	248.515
SD	1.649	0.313	1860.782	926.726	0.607	1.009	4.248	0.506	652.952

Compared to the rhegmatogenous retinal detachment control group, most cytokines concentrations were not statistically significant different, except IL-1β (Mann-Whitney Test: IL-1β P = 0.032; IL-2 P = 0.306; IL-6 P = 0.151; IL-8 P = 0.366; IL-10 P = 0.436; IL-17 P = 0.149; TNF-α P = 0.245; IFN-γ P = 0.297; VEGF P = 0.897) (Figure 3).

### VEGF level in the intra-FCVB fluid measured by ELISA

In the intra-FCVB fluid, the VEGF concentrations of 6 samples (Patient 02, 03, 05, 07, 09, and 11) were measured by ELISA. Among these patients, Patient 02 and 09 showed a high level of VEGF (02: 2925.7 pg/ml and 09: 127.6 pg/ml). These results were in agreement with the Multiplex immunoassay of VEGF concentrations (02: 1864.25 pg/ml and 09: 27.2 pg/ml). There was a small difference between the Multiplex immunoassay and ELISA results. The reason for this was likely that the cytokines concentrations in these samples would decrease with time and were affected by the freeze-thaw cycle. It is notable that we tested the samples by ELISA first. Nevertheless, the measured VEGF levels increased in parallel in these two results. The VEGF concentrations of the remaining four patients were less than 15.6 pg/ml because they were below the range of detection sensitivity.

## Discussion

In this study, the majority of inflammatory immune mediators were detectable at low levels (<10 pg/ml) in most intra-FCVB fluids aspirated from the FCVB-filled eyes at the third month (Table 4). This demonstrated that the FCVB possessed sufficient porosity to allow cytokines to pass through. It was the first time that we found the FCVB have the permeability of biological molecules (proteins) in the human eye. While, the permeability variation of these porous FCVBs was likely reflected in the differences of the detectable cytokine concentrations in the intra-FCVB fluids.

In our previous exploratory clinical trial, every patient was designed to be injected with BSS into the FCVB on the FCVB implantation surgery day. The ingredients of the injected BSS was equivalent, and consisted of glucose, sodium chloride, magnesium sulfate, potassium chloride, calcium chloride, and sodium bicarbonate [22]. Theoretically, no or few proteins including cytokines would be detected in the intra-FCVB fluid.

Why did these cytokines emerge in the intra-FCVB fluid? Our previous study proved that the FCVB had numerous 300 nm mini-apertures on the capsular wall which could potentially serve as a drug delivery system (DDS) to release drugs (dexamethasone sodium phosphate) into an intraocular environment outside the FCVB [23]. Therefore, we wonder whether the intraocular proteins outside the FCVB could pass through the mini-apertures on the capsular wall of the FCVB.

Where did these cytokines come from at the third month? In this clinical trial, a unexpected thing happened that a few point-like opacities were found in intra-FCVB fluids in Patient 02 and 06 on day 11 and day 7 after FCVB implantation surgery. These opacities persisted until the 3th month in these two patients (Figure 2). HE staining showed that these opacities were a few translucent membranes which could well be the cytokines and other proteins. Therefore, these cytokines probably have originated from the transient anterior segment inflammatory reaction in the early post-operative stage in the FCVB-filled eyes, and some of them stayed in FCVB capsules during three months.

From a physical standpoint, the permeability of the FCVB is dependent on two main factors: the FCVB itself and the internal and external environment surrounding the FCVB. In this study, the amount and size of the mini-apertures on the capsular wall varied in every FCVB-filled eye. The internal environment of the FCVB was rendered uniform, as the same BSS was injected into the FCVB on the surgery day. Therefore, the external environment of the FCVB was likely the critical factor in determining the propensity for cytokine passage through the mini-apertures. Apparently, the most powerful driving force was the high concentration gradient of cytokines outside the FCVB happened at the early post-operative anterior segment inflammation (Figure 2). As time went on, the cytokine concentrations would remain relatively constant once equilibration of the cytokine concentrations inside and outside the FCVB occurred.

Why did IL-6, IL-8, and VEGF exist in an exceedingly high level in a few FCVBfilled-eyes (Patient 02, 06, and 09) (Table 4, underlined data)? This might be associated with the severity of the ocular injury in these patients. These patients all suffered from a severe penetrating ocular injury complicated with retinal detachment (Table 1). Patient 02 was a 13 year old boy, who suffered from severe ocular injury and the critical wound repair inflammatory mediators of IL-6 and IL8 would be released. A marked hyphema and severe retinal detachment in this patient indicated serious damage to the blood-ocular barrier and would explain the elevated level of VEGF for this patient. Patient 06, the only female in this study, was a middle-aged woman and the rising IL-6 and IL-8 might be related with a significant fibrinous exudation observed in the anterior chamber during the first postoperative week (Figure 2). Patient 09 was a young man who, prior to the FCVB implantation surgery, received a number of PPV surgeries for the treated eye and was tamponaded with heavy silicone oil. The rising IL-6 might have been induced by residual heavy silicone oil or simply reflected the surgical trauma of multiple intraocular surgeries.

Interestingly, IL-6, IL-8, and VEGF levels were also relatively high compared to other cytokines in the undiluted vitreous fluid of 6 rhegmatogenous retinal detachment eyes (Figure 3). It has been reported that these cytokines (IL-6, IL-8, and VEGF) may have a relationship with retinal detachment. Ricker et al reported that the chemokines (including IL-8) and IL-6 are up-regulated in rhegmatogenous retinal detachment patients with fibrotic membranes and might be involved in the development of postoperative PVR [24]. Another study also showed that an increasing IL-8 and VEGF may be related to the development of PVR [25]. In this study, these patients (Patient 02, 06, and 09) all suffered a severe retinal detachment complicated with severe PVR. Therefore, these high levels of cytokines in these select intra-FCVB fluid samples may reflect the intrinsic severity of the ocular disease itself, instead of the material of the FCVB.

In our previous study using rabbit eyes, the FCVB proved to be a suitable DDS for the mechanically sustained release chemical drugs (dexamethasone, levofloxacin, 5-fluorouracil) [23], [26]–[28]. This study first discovered that the porous FCVB possesses favorable permeability of proteins in the human eye. Therefore, it hopefully provides valuable information as to the use of FCVB in the development of a DDS for the release biological drugs (eg., bevacizumab, nerve growth factor) for human ophthalmological clinical applications.

In conclusion, the cytokines that emerged in the intra-FCVB fluid probably originated from early post-operative anterior segment inflammation. The results of this study suggest that the FCVB itself possessed sufficient porosity to allow protiens to pass through. It was the first time that we found FCVB have the permeability of biological molecules in human eye.

## Supporting Information

Text S1 **Evaluation of the Flexibility, Efficacy, and Safety of a Foldable Capsular Vitreous Body in the Treatment of Severe Retinal Detachment.**
(PDF)
