# Supplementary material for: Comprehensive Analysis of Inflammatory Immune Mediators of the Intraocular Fluid Aspirated from the Foldable Capsular Vitreous Body Filled-Eyes
Source: PLoS One. 2012 Oct 1;7(10):e46384. doi: 10.1371/journal.pone.0046384 (PMC3462176; doi:10.1371/journal.pone.0046384)
Supplement: Text S1 — Evaluation of the Flexibility, Efficacy, and Safety of a Foldable Capsular Vitreous Body in the Treatment of Severe Retinal Detachment. (PDF) [file pone.0046384.s001.pdf]

Evaluation of the Flexibility, Efficacy, and Safety of a Foldable Capsular Vitreous Body in the Treatment of Severe Retinal Detachment

Xiaofeng Lin,[1,2] Jian Ge,[1,2] Qianying Gao,[1] Zhenfang Wang,[1] Chongde Long,[1] Liwen He,[1] Yaqin Liu,[1] and Zhaoxin Jiang[1]

Purpose. To determine the flexibility, efficacy, and safety of a novel foldable capsular vitreous body (FCVB) in the treatment of severe retinal detachment in human eyes.

Methods. The study involved 11 patients with 11 severe retinal detachments. A standard three-port pars plana vitrectomy was performed, and the FCVB was triple-folded and implanted into the vitreous cavity. Balanced salt solution was then injected into the capsule of the FCVB to support the retina. The treated eyes were examined by ophthalmoscopy, fundus photography, and tonometry during a 3-month implantation period. B-scan ultrasonography, optical coherence tomography (OCT), ultrasound biomicroscopy (UBM), and electroretinogram (ERG) were also performed. The FCVB was removed and examined in the laboratory at the end of the 3-month treatment time.

Results. The FCVB was easily implanted into the vitreous cavity through a 3-mm incision and was easily removed through a 2-mm scleral incision. Retinal reattachment was found in 8 (73%) of 11 eyes at the end of the 3-month treatment time. The fundus, B-scan, and OCT showed that the FCVB was well distributed in the vitreous cavity and evenly supported the retina. IOP and visual acuity in the FCVB-treated eyes did not show a significant difference when compared with the preoperative measurements. UBM showed that the FCVB smoothly contacted but did not crush the ciliary body. Laboratory examinations showed no significant inflammatory cells in the balanced salt solution, no decrease in spectral transmittance, and no blocking of tiny apertures from the FCVB after a 3-month implantation period.

Conclusions. The FCVB was shown to be flexible, effective, and safe as a vitreous substitute over a 3-month implantation time. (ClinicalTrials.gov number, NCT00910702.) (*Invest Ophthalmol Vis Sci.* 2011;52:374–381) DOI:10.1167/iovs.10-5869

The vitreous body is a transparent, gelatinoid structure that occupies four-fifths of the volume of the eye. It consists of approximately 99% water and 1.0% inorganic salts, organic lipids, and hyaluronan, which can maintain a certain spatial relationship with dipolar water molecules.[1] The physiological function of the vitreous body involves support of adjacent posterior segment structures, provision of ocular refractive media, and provision of a cell barrier to inhibit cell migration from the retina to the vitreous cavity.[2]

Pars plana vitrectomy (PPV), with artificial vitreous substitutes, can restore vision in many patients, such as those affected by proliferative diabetic retinopathy (PDR), proliferative vitreoretinopathy (PVR), traumatic PVR, and endophthalmitis—patients whose ocular conditions would previously have been regarded as hopeless.[3–9]

Several artificial vitreous substitutes (e.g., silicone oil, heavy silicone oil, and hydrogels) have been adopted.[10–19] Among these, silicone oil, reported by Cibis in 1962, is one of the most common ones used in the clinic. Although it has saved many patients from blindness over the past 50 years, it still causes concomitant complications, such as glaucoma, cataracts, corneal degeneration, and emulsification. Moreover, the low-density silicone oil cannot support the inferior retina, and it induces a hyperopic shift in the optical system. Although silicone oil is a relatively safe vitreous substitute, it may flow into the anterior chamber, subretina, or even out of the eyeball and result in difficult removal because of the free fluid characteristics within the eye. Therefore, despite a half century of effort to replace the vitreous body of the eye, an ideal and permanent vitreous body has yet to be found.[20,21]

In our previous studies, we proposed a novel strategy to replace the natural vitreous body: a foldable capsular vitreous body (FCVB).[22–24] The FCVB consists of a thin vitreous-shaped capsule with a tube-valve system made with computer and industrial technology. After the folded body is installed in the eye, balanced salt solution is then injected into the capsule, inflating it to support the retina. The tube–valve system allows control of the intraocular pressure (IOP).[22] Interestingly, FCVB changes the refraction very little when compared with silicone oil and heavy silicone oil, based on the Gullstrand-Emsley and Liou-Brennan schematic eyes.[23] Reports from the State Food and Drug Administration in China show that the FCVB has good mechanical, optical, and biocompatible properties (No. G20080656).[24] The FCVB is composed of a liquid silicone rubber, which is a non-toxic and stable material.[24] In regard to its mechanical properties, the liquid silicone rubber has suitable hardness and high strain capability that allows the 60-μm thin FCVB to stretch its capsule to evenly and gently support the detached retina. Optical properties indicate that the material has high light transmission and laser irradiation stability.[24] Because the FCVB has never been used in human eyes anywhere in the world, we conducted an exploratory study at Zhongshan Ophthalmic Center to evaluate its flexibility, safety, and efficacy and to provide experiences and standards for a multiple-center clinical trial.

From the [1]State Key Laboratory of Ophthalmology, Zhongshan Ophthalmic Center, Sun Yat-sen University, Guangzhou, China.

[2]These authors contributed equally to the work presented here and should therefore be regarded as equivalent authors.

Supported by Grant 2009AA2Z404 from the National High-tech R&D Program of China (863 Program).

Submitted for publication May 12, 2010; revised July 22 and August 4, 2010; accepted August 8, 2010.

Disclosure: **X. Lin**, None; **J. Ge**, None; **Q. Gao**, None; **Z. Wang**, None; **C. Long**, None; **L. He**, None; **Y. Liu**, None; **Z. Jiang**, None

Corresponding author: Qianying Gao, Xianlie Road 54, Guangzhou, Guang Dong, China 510060; gaoqy@mail.sysu.edu.cn.

Investigative Ophthalmology & Visual Science, January 2011, Vol. 52, No. 1
Copyright 2011 The Association for Research in Vision and Ophthalmology, Inc.

Patients and Methods

Study Design

The study protocol was reviewed and approved by the Sun Yat-sen University Medical Ethics Committee (Zhongshan Ophthalmic Center Medical Ethics [2009] No. 07). The clinical trials adhered strictly to the principles of The World Medical Association Declaration of Helsinki and were registered with the National Institutes of Health and the Chinese Clinical Trial Registry (ChiCTR-TNC-00000396). All patients gave written informed consent.

To be included in the study, patients had to have a severe retinal detachment that could not be easily reattached with silicone oil tamponade, such as posterior scleral ruptures with large disruptions of the retina or severe scleral ruptures with retinal and choroidal detachments, or they had to have rigid retinal redetachments or inferior holes that occurred after silicone or heavy oil tamponade had been attempted. An independent committee of experts confirmed that the detached retina could not be easily reattached with silicone oil. The surgeon who was to install the FCVB again confirmed the committee's conclusion during the placement. If the surgeon believed that the detachment could be treated with oil tamponade, he could stop the procedure and deny the patient FCVB treatment.

Excluded from participating were patients with serious heart, lung, liver, or kidney dysfunction; serious eye inflammation; only one eye; suitably silicone oil-filled eyes; or diseases that made them unsuitable for inclusion.

Study Patients

Between May 2009 and January 2010, 11 patients (11 eyes) were enrolled in the study. The demographic and ocular characteristics of the patients at baseline examination are shown in Table 1. The mean age of the patients was 27.5 years (13–53 years), and 10 (90.9%) of the 11 patients were male. Of the 11 eyes, 1 (9.1%) had a severe ocular rupture, 6 (54.5%) had penetrating injuries, and 4 (36.4%) had contusion of the eyeball combining large defects of the retina or choroid. Of these eyes, 4 (36.4%) had failed to respond to silicone oil tamponade, 2 (18.2%) failed treatment with C3F8, and 1 (9.1%) failed to respond to heavy silicone oil.

Study Treatment

Intervention procedures consisted of vitrectomy, FCVB (Guangzhou Vesber Co., Ltd., Guangzhou, China) insertion, and FCVB removal. A standard three-port pars plana vitrectomy was performed, and membrane peeling, retinotomy, and relaxing retinectomy were added if necessary. After a 3×1-mm scleral incision was created, the FCVB was triple folded and inserted into the vitreous cavity without air–fluid exchange. Approximately 4.0 mL of balanced salt solution was injected into the FCVB capsule through the valve with a 5-mL syringe. The infusion fluid in the eye escaped through the scleral incision when the capsular bag was inflated to the point that it was supporting the retina. The volume of the air in the eye was replaced by balanced salt solution through an infusion cannula when the capsular bag was deflated. The tube and valve were subsequently fixed onto the scleral surface. Ocular examinations were performed at baseline, 3 months after FCVB inser-

Table 1. Demographic and Ocular Characteristics of Patients at Baseline Examination

Patient	Age (y)	Sex	History of Surgery	Diagnosis
01	53	Male	Scleral wound exploration and repair + eyelid skin laceration suture	Ocular contusion (OD) Scleral and corneal laceration; hyphema; iridodialysis; vitreous hemorrhage; choroidal detachment; retinal detachment
02	13	Male	Corneal wound suturing + formation of anterior chamber; PPV + C3F8	Penetrating ocular injury (OS) Penetrating corneal trauma; hyphema; iridocoloboma; aphakic eye; vitreous opacity; retinal detachment
03	19	Male	Corneal wound suturing; lensectomy + PPV + silicone oil	Ocular contusion (OD) Corneal laceration; iridocoloboma; aphakic eye; silicone oil eye; retinal detachment
04	38	Male	Corneal wound suturing	Penetrating ocular injury (OD) Penetrating corneal trauma; traumatic cataract; retinal detachment
05	13	Male	Corneal wound suturing; PPV + silicone oil	Ocular contusion (OD) Corneal laceration; iridocoloboma; aphakic eye; silicone oil eye
06	30	Female	Corneal wound suturing; lensectomy + PPV; encircling scleral buckling + PPV + silicone oil	Penetrating ocular injury (OD) Penetrating corneal trauma; aphakic eye; silicone oil eye; retinal detachment
07	43	Male	Scleral wound exploration and repair + eye skin laceration suture	Ocular contusion (OS) Scleral laceration; traumatic cataract; vitreous hemorrhage; choroidal detachment; retinal detachment
08	18	Male	Corneal wound suturing	Ocular contusion (OD) Corneal laceration; aphakic eye; vitreous hemorrhage; choroidal detachment; retinal detachment
09	25	Male	Intraocular foreign body extraction; PPV; PPV + C3F8; PPV + heavy silicone oil	Penetrating ocular injury (OS) Penetrating scleral trauma; traumatic cataract; heavy silicone oil eye; retinal detachment
10	19	Male	Corneal wound suturing; PPV + scleral cryosurgery + silicone oil; silicone oil removal	Penetration ocular injury (OS) Corneal leucoma; iridocoloboma; aphakic eye; retinal detachment
11	30	Male	Corneoscleral trauma suturing	Penetration ocular injury (OS) Penetrating corneoscleral trauma; traumatic cataract; choroidal detachment; retinal detachment

tion, and 3-months after removal and included determination of visual acuity with Snellen eye charts and IOP with Goldmann applanation tonometry (BM 900; Haag–Streit, Bern, Switzerland), slit lamp biomicroscopy, direct ophthalmoscopy, and fundus photography (TRC-50EX; Topcon, Tokyo, Japan). B-scan ultrasound (CineScan A/B; Quantel Medical, Bozeman, MT) was performed at baseline; at 2, 4, and 8 weeks and 3 months after FCVB insertion; and at 3 months after removal. A-scan ultrasound (CineScan A/B; Quantel Medical), optical coherence tomography (OCT, Visante; Carl Zeiss Meditec, Dublin, CA), ultrasound biomicroscopy (UBM, SW-3200 Kinscan; Suoer, Tianjin, China), noncontact specular microscopy (SP-3000P; Topcon, Tokyo, Japan), and electroretinogram (ERG, UTAS-3000 ERG System; LKC, Gaithersburg, MD) were performed at baseline and 3months after implantation. The postoperative IOP could be restored by injection of balanced salt solution through the valve with a 2-mL syringe. Then, the FCVB was removed, and the eye was filled with balanced salt solution (n = 4 eyes), inert gas (n = 2 eyes), or silicone oil (n = 5 eyes), depending on the condition of the eye, and was observed for 3 months after removal.

Outcome Measures

The primary outcome measure was the complete retinal reattachment rate determined by B-scan ultrasound at the end of the 3-month implantation time. If the fundus was clear, OCT was also performed.

The secondary outcome measures included visual acuity, IOP, and axial length, as determined by the Snellen chart, Goldmann applanation tonometry, and A-scan, respectively. Visual acuity was graded

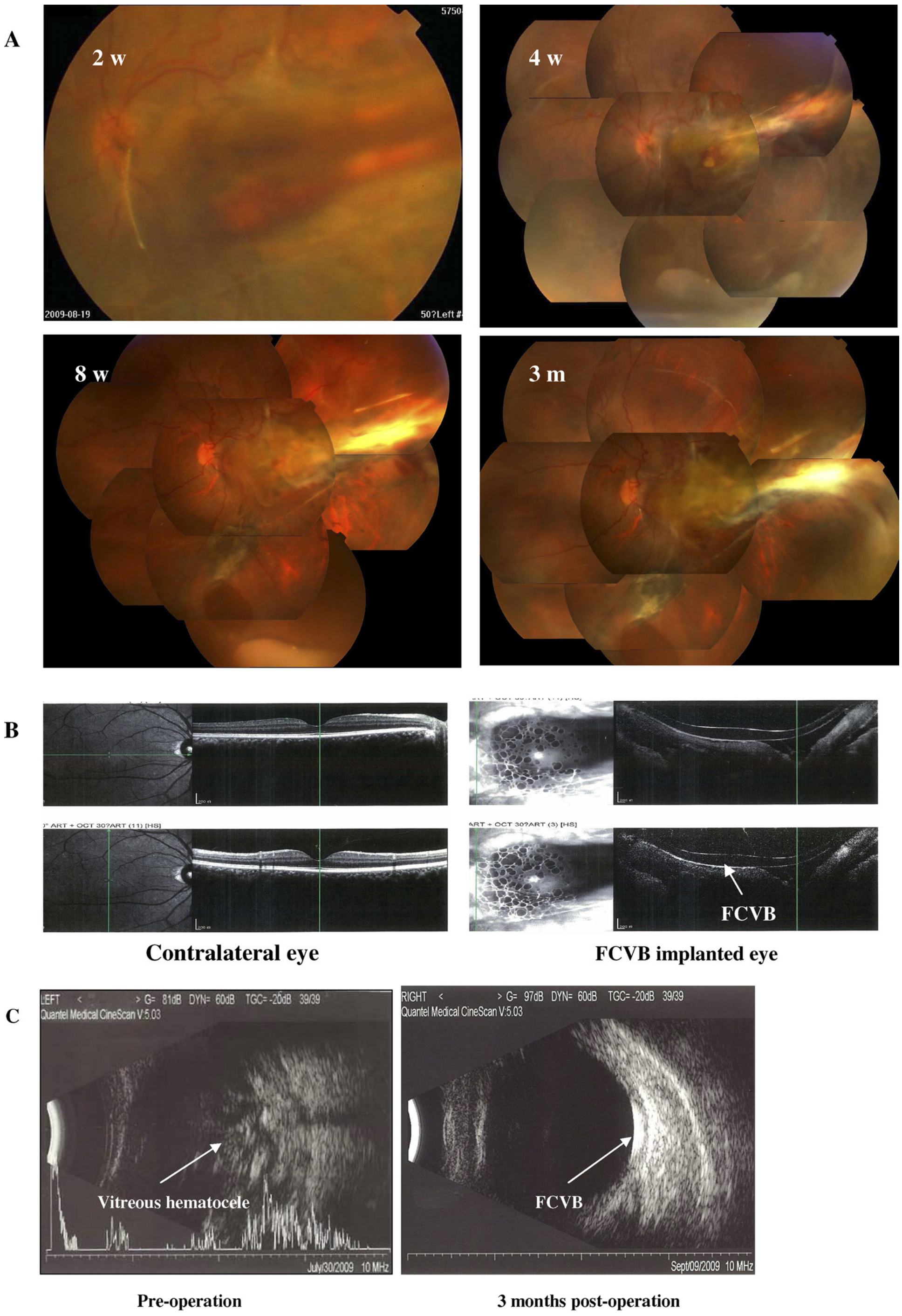

FIGURE 1. Results of 3-month FCVB implantation in patient 7. **(A)** The fundus of the FCVB-filled eyes was clearly visible, the retina was reattached, and the FCVB capsule supported the retina and eye well, without any wrinkles during the 3-month implantation time. **(B)** OCT indicated that the 60-μm-thick capsular membrane could evenly support the retina. **(C)** The B-scan showed a capsule-like arc reflective signal indicating support of the retina.

according to the following system: no light perception, 0; light perception 1; hand motion perception, 2; finger count perception, 3; ≥0.05 acuity, 4; and ≥0.1 acuity, 5. The preoperation vision acuities were very low, scoring from 0.5 to 2.

All photographically derived outcomes were determined by independent grading of retinal photographs, performed by graders at the Fundus Photograph Reading Center who were unaware of treatment assignments.

Laboratory Examinations

Because of the 300-nm tiny apertures in the capsule of the FCVB, we tested whether the level of balanced salt solution in the FCVB changed during the 3-month implantation period. The solution was withdrawn with a 5-mL syringe before the removal of the FCVB and subjected to pathologic hematoxylin and eosin (HE) staining. The FCVB was then removed and tested with spectral transmittance and a scanning electron microscope. Spectral transmittance tests were carried out according to ISO 11979-2-1999.[25] Spectral transmittance is the ratio of the transmitted radiant flux (regular and diffuse) to the incident radiant flux when a parallel beam of monochromatic radiation of a given wavelength passes through a specimen. The capsule of the FCVB was cut into the appropriate size and cleaned, coated with gold, and fixed on a specimen stub. The image of the specimen surface was captured on a scanning electron microscope.

Statistical Analysis

Bonferroni multiple comparisons were used to compare preoperative IOP and visual acuity with IOP and visual acuity at each time point after FCVB implantation. A paired-samples test was used for comparison of axial length, corneal edema, and corneal endothelial cells between baseline and 3 months after implantation. The Wilcoxon signed-rank test was used for conjunctival congestion, keratic precipitate, and aqueous flare between baseline and 3 months after implantation. Statistical significance was set at the 5% level.

Results

Flexibility Studies

The FCVB was easily inserted into the vitreous cavity through a 3-mm incision and was easily removed through a 2-mm scleral incision (Supplementary Movies S1 and S2, http://www.iovs.

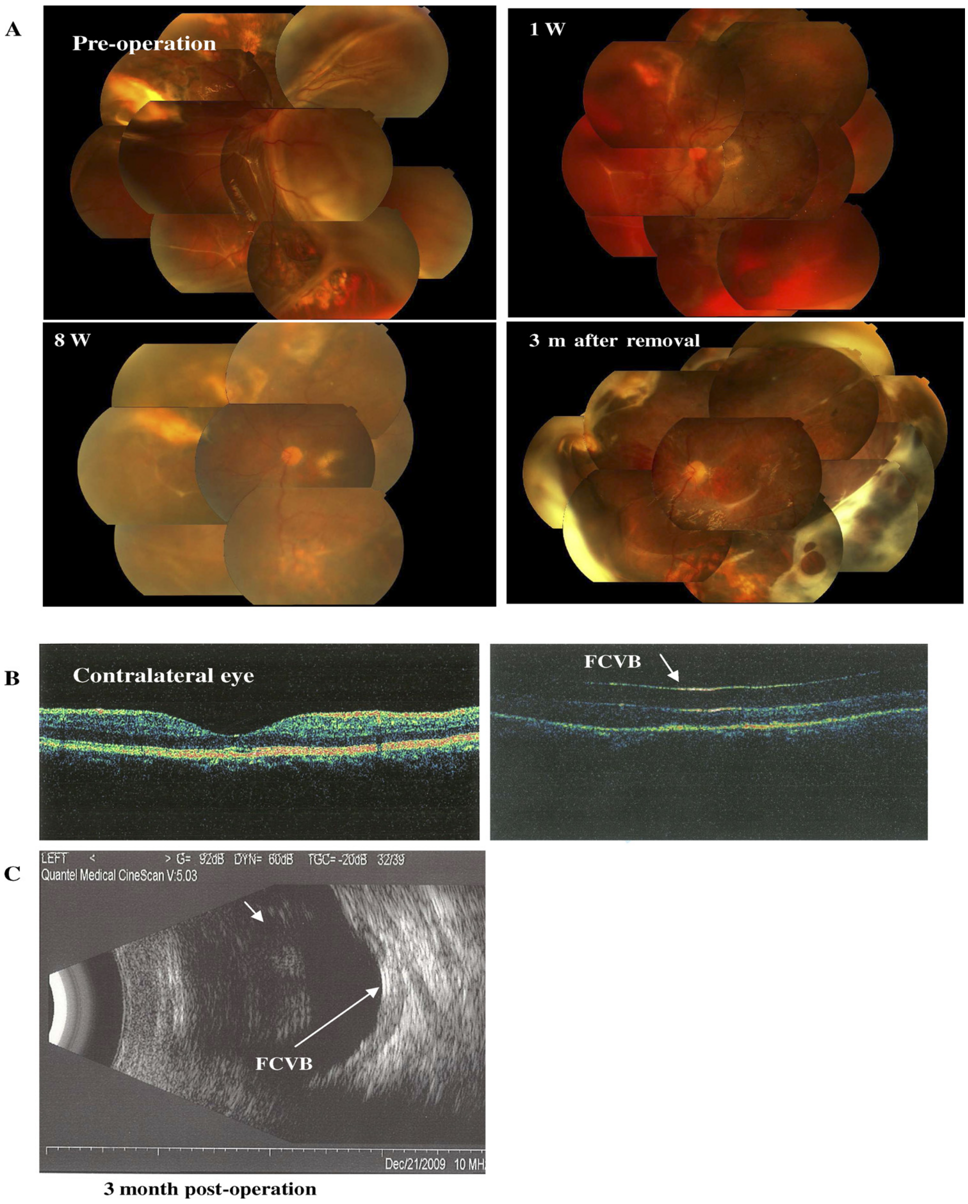

Figure 2. A 3-month FCVB implantation in patient 9. (**A**) The retina had redetached after heavy silicone oil tamponade. The retina was reattached, and the FCVB capsule supported the retina and eye during the 3-month observation time (cataract occurred at 3 months). The retina was still reattached at 3 months after cataract extraction and FCVB removal. (**B**) OCT indicated that the capsular membrane provided even support for the retina. (**C**) The B-scan showed a capsulelike arc reflective signal from the retina.

org/lookup/suppl/doi:10.1167/iovs.10-5869/-/DCSupplemental). During the implantation time, IOP was regulated with balanced salt solution injected or removed through a 2-mL syringe.

Efficacy Evaluations

Primary and Secondary Endpoints. Retinal reattachments were found by B-scan in 8 (73%) of the 11 eyes at the end of the 3-month treatment time. A good profile of the eye was visible in the FCVB-implantation eye. The fundus of the FCVB-filled eyes was clearly visible and the FCVB was well distributed in the vitreous cavity at 3 days. The retina was reattached and the capsule of the FCVB supported it and the whole eye well, without any wrinkles forming in the retina during the 3-month implantation period (Figs. 1A, 2A). OCT indicated that the 60-μm-thick capsular membrane evenly supported the retina. In addition, a very thin gap between the film and the retina prevented sticking (Figs. 1B, 2B). The B-scan showed a capsule-like arc reflective signal from the retina (Fig. 1C, 2C). Retinal reattachments were observed in 8 and in 5 of 8 eyes at 1 and 3 months after FCVB removal, respectively. Based on these data, it appears that the FCVB was well distributed in the vitreous cavity and supported the retina evenly.

IOP is essential to sustain the shape of the eye. It seemed that the IOP at the 3-month implantation time was slightly lower than at baseline. However, the IOPs at each time point in the FCVB-treated eyes did not show a significant difference, except at 4 weeks ($P = 0.015$) during the 3-month implantation and 3 months after capsule removal ($P > 0.05$, Fig. 3A). Similarly, the scores for visual acuity at each time point did not show a significant difference from those at baseline ($P > 0.05$, Fig. 3B). Compared with preoperative data, no difference was found for axial length at 3 months in the FCVB-treated eyes (Fig. 3C; $P > 0.05$). The ERG could not be recorded before or after FCVB implantation.

Safety Evaluation. There was a slight conjunctival hyperemia by day 7 after surgery in the FCVB-filled eyes. No serious complications (e.g., corneal keratopathy or intraocular inflammation) were observed. There was no statistically significant conjunctival congestion, corneal edema, keratic precipitate, or aqueous flare at baseline or at 3 months after implantation (Fig. 4). The Wilcoxon signed rank test was used to test for conjunctival congestion ($t = 2.50$, $P = 0.317$), keratic precipitate ($t = 0$, $P = 0.083$), and aqueous flare ($t = 0$, $P = 0.102$), and the paired-samples test was used to test for corneal edema ($t = 0.314$, $P = 0.763$).

In two cases, anterior chamber hyphema was observed, owing to the relief of epiretinal traction during the surgery. UBM showed that the FCVB smoothly contacted but did not crush the ciliary body. No edema or atrophy of the ciliary body was observed (Fig. 5). There were no statistically significant corneal endothelial cell counts at baseline or after 3 months of implantation (paired samples test: $t = 0.487$, $P = 0.674$). Laboratory examinations showed that there were a few translucent membranes in the balanced salt solution in the 3-month FCVB-implanted eyes, and there were no inflammatory cells, bacteria, or fungi present. The spectral transmittance test of the FCVBs revealed that 93% of the implants were observed before implantation and 91% after removal. Scanning electron microscope images of the capsule of the FCVB show that the 300-nm apertures were still open after the 3-month implantation period.

Adverse Events. Adverse events were defined as unbearable foreign body sensations, abnormal bleeding, severe inflammation, endophthalmitis, and sympathetic ophthalmia. No adverse events were observed during the study.

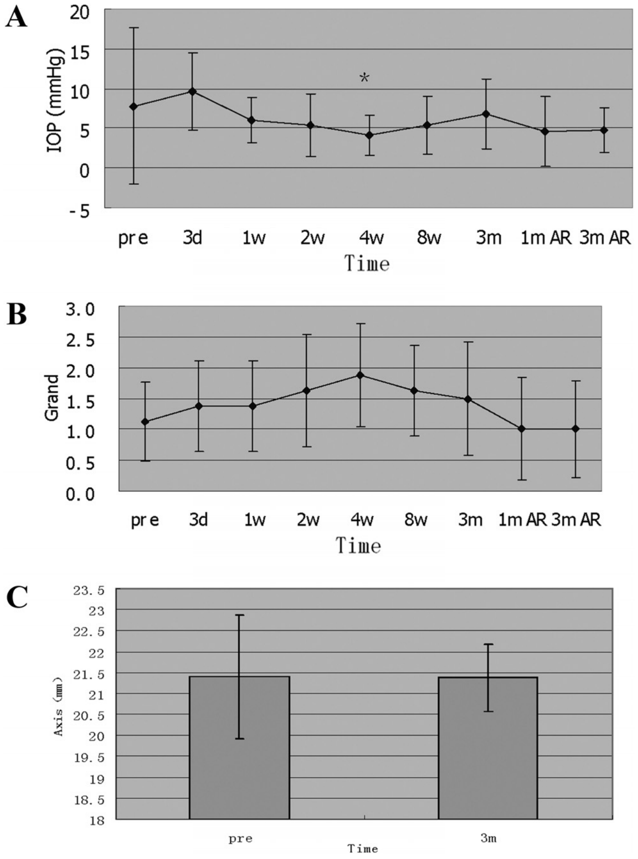

Figure 3. Dynamic changes in IOP (**A**), visual acuity scores (**B**), and axial length (**C**) during the 3-month implantation period. At each time point after FCVB implantation, IOPs did not show a significant difference compared with of preoperative IOPs (except at 4 weeks), during a 3-month implantation and a 3-month follow-up period. Similarly, the visual acuity scores at each time point after FCVB implantation did not show a significant difference compared with those at baseline. Compared to preoperative data, no difference was found for axial length at 3 months in the FCVB-treated eyes. AR, after removal.

Discussion

In the present study, we used a new strategy and product to replace the natural vitreous by FCVB and first demonstrated that the FCVB can restore the structure and partial function of the vitreous in the treatment of severe retinal detachment in human eyes. The present study showed that FCVB is a flexible, effective, and safe artificial vitreous for treatment of severe retinal detachment during a 3-month implantation time.

Although the Supplementary Movies S1 and S2 (http://www.iovs.org/lookup/suppl/doi:10.1167/iovs.10-5869/-/DCSupplemental) showed that the FCVB was easily inserted and removed without any conglutination with the retina, an auxiliary tool for easy implantation (e.g., an intraocular lens) should be designed. In addition, IOP can be modulated through the valve with a syringe. To our knowledge, there are no other vitreous substitutes that have such a facilitative function.

Silicone oil tamponade is designed to support the retina by surface tension.[10–13] Because the oil is lighter than water, it is unable to adequately support the inferior detached retina, which can be supported by heavy silicone oil.[14,15] However, the FCVB was designed to support the retina and the eye by providing a solid arc. The inferior detached retina was effectively repaired by the FCVB, as shown in Figures 1 and 2. After

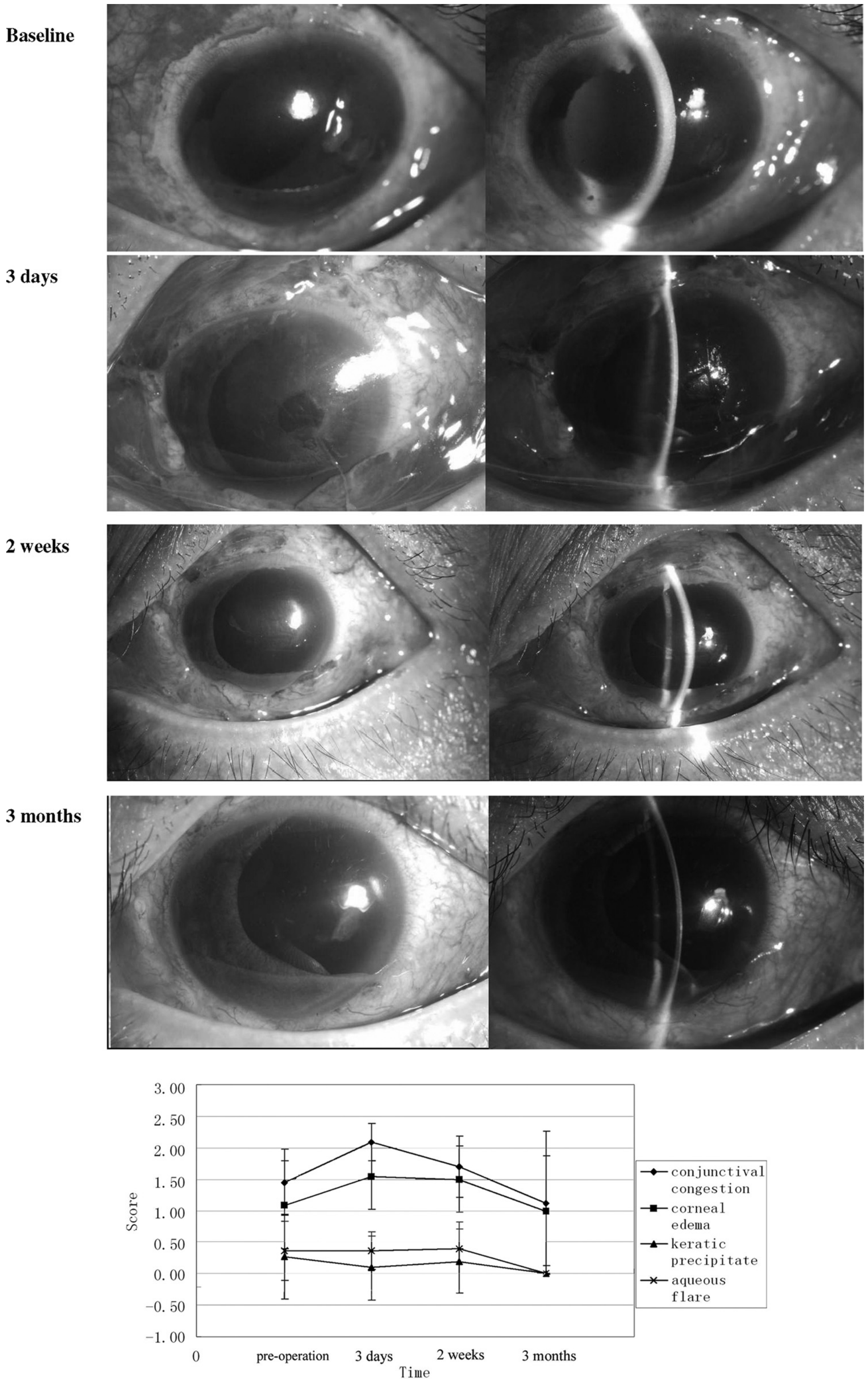

Figure 4. Anterior segment photography at baseline, 3 days, 2 weeks, and 3 months after FCVB implantation surgery. There was no statistically significant conjunctival congestion, corneal edema, keratic precipitate, or aqueous flare at baseline or 3 months after FCVB implantation. The Wilcoxon signed-rank test was used to test for conjunctival congestion ($t = 2.50$, $P = 0.317$), keratic precipitate ($t = 0$, $P = 0.083$), and aqueous flare ($t = 0$, $P = 0.102$), whereas the paired samples t-test was used to test for corneal edema ($t = 0.314$, $P = 0.763$). The vertical arc is a flash artifact.

3 months of FCVB implantation, 8 of the 11 retinas were reattached; leakage of the FCVB caused failure in three eyes. We have revised the production method of the FCVB and solved the problem. At 3 months after FCVB removal, retinas in 5 of 11 eyes were still reattached and 3 of 11 were redetached. Among the three reattached retinas, two were treated with inert gas tamponade after FCVB removal. The redetachment was probably related to the prior severe trauma or repeated surgeries from which the retina had not fully recovered. Current data indicate that the severest retinal detachments, as mentioned above, may need much longer implantation of the FCVB.

Three months after FCVB implantation, we found that the structure and partial function of the eyes was gradually restored. As shown in Figure 3, visual acuity and IOP in postoperative FCVB eyes did not increase significantly when compared with the preoperative values. The lack of improvement is probably attribut-

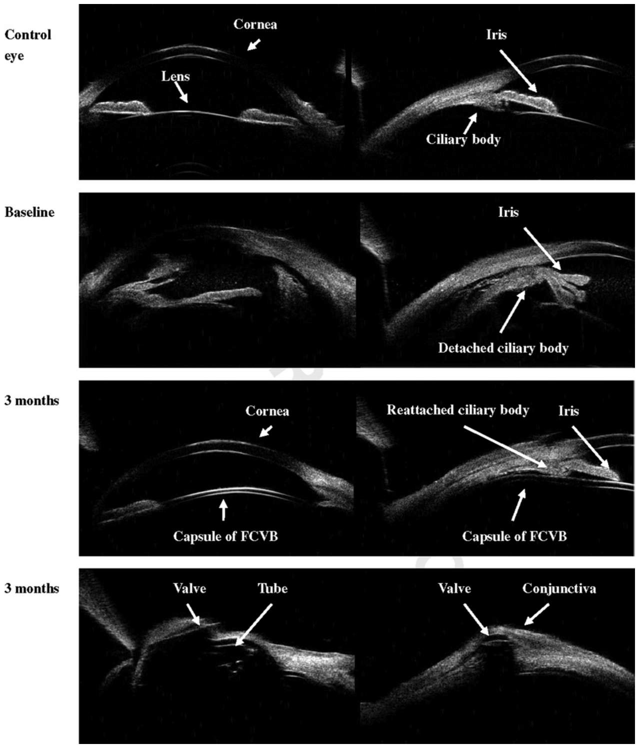

FIGURE 5. UBM at baseline and 3 months after FCVB implantation. UBM showed that the FCVB smoothly contacted and did not crush the ciliary body. No edema or atrophy of the ciliary body was observed.

able to severe conditions such as macular scarring or choroidal detachment and multiple previous surgeries before FCVB implantation. Because of image distortion and nonconductivity, B-scan and ERG were not successful in the silicone oil–filled eyes. Fortunately, although the ERG was still not recordable in the FCVB-treated eyes, the B-scan successfully showed an arc highlight (Figs. 1C, 2C). From these data, we can conclude that the FCVB showed good efficacy during the 3-month implantation time in this exploratory clinical trial.

The FCVB capsule can provide the detached retina with a platform for formation of a flat scar and a barrier to block cell migration from the retina into the vitreous cavity. In the rabbit model of PVR, we found that the FCVB very closely mimicked the eye's morphology and restored physiological functions such as support, refraction, and cellular barriers during a 3-month observation period (data not shown).

There was no obvious inflammation in any of the eyes after implantation. Although hemorrhage was observed in the anterior chamber in three eyes, it was not related to the FCVB. UBM showed that the FCVB did not crush the ciliary body. There was no obvious difference between the FCVB's spectral transmittance before implantation and after removal. Laser irradiation stability tests demonstrated that the FCVB could sustain a 1500-mW, 0.2-second, 532-nm green laser. Retinal photocoagulation with a 200-mW laser output after FCVB implantation was successfully performed in two patients in this study. In FCVB-treated eyes, due to the constraints of the capsule, it was impossible for the liquid in the FCVB to enter the anterior chamber, whereas in silicone oil–filled eyes, the oil can migrate into the anterior chamber (11%–49% of eyes)[26] and induce complications such as glaucoma and corneal keratopathy. In addition, in our study, the patients were able to maintain a normal position, regardless of the time of day, whereas patients with eyes filled with silicone oil must maintain a prone position, which can become very uncomfortable.

In the rabbit study, a silicon chip was implanted under the retina. Histologic studies and TdT-mediated dUTP nick-end labeling (TUNEL) showed no structural abnormalities or apoptosis in the retina over a 1-year observation period (data not shown).

Because of the 300-nm tiny apertures in the capsule, the FCVB can sustainably and mechanically release dexamethasone sodium phosphate and be used as an intravitreal drug delivery system (DDS), in addition to serving as a vitreous substitute.[27] Therefore, the FCVB is a new potential approach for combining vitreous substitutes and drug treatments such as antibiotics, anti-proliferation agents, and vascular endothelial growth factor antagonists.

Because the greatest risk of retinal redetachment occurs 2 months after surgery, the FCVB was designed to be implanted into the eyes for 3 months. However, only 5 of 11 eyes stayed reattached at 3 months after FCVB removal, although the severity of the ocular condition in the current patients was much greater than that commonly found. Future study will focus on long-term implantation and observation of the FCVB as a permanent endotamponade. In addition, we designed a fovea lentis in the FCVB (radius = 6.0 mm) to match the lens,[24] but the effects of FCVB on the lens should be further evaluated, since 10 of 11 eyes were aphakic and only one eye was phakic.

In conclusion, FCVB was shown to be a flexible, effective, and safe vitreous substitute over a 3-month implantation period. Further multiple-center clinical trials are in progress in China to ascertain the efficacy and safety of the FCVB as a novel vitreous substitute.

Acknowledgments

The authors thank the patients and their families for their continuous support of the study; the medical, operating-room, anesthesia, and nursing staff at Zhongshan Ophthalmic Center; and the staff in the Division of Ophthalmology.

References

1. Green WR, Sebag J. Vitreoretinal interface. In: Ryan SJ, eds. *Retina*. 4th ed. Philadelphia: Elsevier-Mosby; 2006;1921–1989.
2. Le Goff MM, Bishop PN. Adult vitreous structure and postnatal changes. *Eye.* 2008;22:1214–1222.
3. Castellarin A, Grigorian R, Bhagat N, Del Priore L, Zarbin MA. Vitrectomy with silicone oil infusion in severe diabetic retinopathy. *Br J Ophthalmol.* 2003;87:318–321.
4. Karel I, Kalvodová B. Long-term results of pars plana vitrectomy and silicone oil for complications of diabetic retinopathy. *Eur J Ophthalmol.* 1994;4:52–58.
5. Pastor JC. Proliferative vitreoretinopathy: an overview. *Surv Ophthalmol.* 1998;43:3–18.
6. Quiram PA, Gonzales CR, Hu W, et al. Outcomes of vitrectomy with inferior retinectomy in patients with recurrent rhegmatogenous retinal detachments and proliferative vitreoretinopathy. *Ophthalmology.* 2006;113:2041–2047.
7. Yoon YH, Lee SU, Sohn JH, Lee SE. Result of early vitrectomy for endogenous Klebsiella pneumoniae endophthalmitis. *Retina.* 2003;23:366–370.
8. Karel I, Michalicková M. Pars plana vitrectomy in the pediatric population: indications and long-term results. *Eur J Ophthalmol.* 1999;9:231–237.
9. Boscia F, Furino C, Prascina F, et al. Combined surgical ablation and intravitreal triamcinolone acetonide for retinal angiomatous proliferation. *Eur J Ophthalmol.* 2005;15:513–516.
10. Cibis PA, Becker B, Okun E, Canaan S. The use of liquid silicone in retinal detachment surgery. *Arch Ophthalmol.* 1962;68:590–599.
11. The Silicone Study Group. Vitrectomy with silicone oil or sulfur hexafluoride gas in eyes with severe proliferative vitreoretinopathy: results of a randomized clinical trial. Silicone Study Report 1. *Arch Ophthalmol.* 1992;110:770–779.

12. Azen SP, Scott IU, Flynn HW Jr, et al. Silicone oil in the repair of complex retinal detachments: a prospective observational multicenter study. *Ophthalmology.* 1998;105:1587–1597.
13. Scott IU, Flynn HW Jr, Murray TG, et al. Outcomes of complex retinal detachment repair using 1000- vs 5000-centistoke silicone oil. *Arch Ophthalmol.* 2005;123:473–478.
14. Bhisitkul RB, Gonzalez VH. "Heavy oil" for intraocular tamponade in retinal detachment surgery. *Br J Ophthalmol.* 2005;89:649–650.
15. Mackiewicz J, Mühling B, Hiebl W, et al. In vivo retinal tolerance of various heavy silicone oils. *Invest Ophthalmol Vis Sci.* 2007;48:1873–1883.
16. Hong Y, Chirila TV, Fitton JH, Ziegelaar BW, Constable IJ. Effect of crosslinked poly (1-vinyl-2-pyrrolidinone) gels on cell growth in static cell cultures. *Biomed Mater Eng.* 1997;7:35–47.
17. Hong Y, Chirila TV, Vijayasekaran S, et al. Biodegradation in vitro and retention in the rabbit eye of crosslinked poly (1-vinyl-2-pyrrolidinone) hydrogel as a vitreous substitute. *J Biomed Mater Res.* 1998;39:650–659.
18. Maruoka S, Matsuura T, Kawasaki K, et al. Biocompatibility of polyvinylalcohol gel as a vitreous substitute. *Curr Eye Res.* 2006;31:599–606.
19. Lam RF, Cheung BT, Yuen CY, et al. Retinal redetachment after silicone oil removal in proliferative vitreoretinopathy: a prognostic factor analysis. *Am J Ophthalmol.* 2008;145:527–533.
20. Colthurst MJ, Williams RL, Hiscott PS, Grierson I. Biomaterials used in the posterior segment of the eye. *Biomaterials.* 2000;21:649–665.
21. Soman N, Banerjee R. Artificial vitreous replacements. *Biomed Mater Eng.* 2003;13:59–74.
22. Gao Q, Mou S, Ge J, et al. A new strategy to replace the natural vitreous by a novel capsular artificial vitreous body with pressure-control valve. *Eye.* 2008;22:461–468.
23. Gao Q, Chen X, Ge J, et al. Refractive shifts in four selected artificial vitreous substitutes based on Gullstrand-Emsley and Liou-Brennan schematic eyes. *Invest Ophthalmol Vis Sci.* 2009;50:3529–3534.
24. Liu Y, Jiang Z, Gao Q, et al. Technical standards of foldable capsular vitreous body regarding mechanical, optical and biocompatible properties. *Artif Organs.* 2010;34:836–845.
25. International Organization for Standardization. ISO 11979-2:1999: Ophthalmic implants: intraocular lenses. Part 2: Optical properties and test methods. Geneva, Switzerland: ISO; 1999.
26. Honavar SG, Goyal M, Majji AB, et al. Glaucoma after pars plana vitrectomy and silicone oil injection for complicated retinal detachments. *Ophthalmology.* 1999;106:169–176.
27. Liu Y, Ke Q, Chen J, et al. Dexamethasone sodium phosphate sustained mechanical release in the foldable capsular vitreous body. *Invest Ophthalmol Vis Sci.* 2010;51:1636–1642.
